# Supplementary material for: The diel disconnect between cell growth and division in Aureococcus is interrupted by giant virus infection
Source: Front Microbiol. 2024 Aug 21;15:1426193. doi: 10.3389/fmicb.2024.1426193 (PMC11371579; doi:10.3389/fmicb.2024.1426193)
Supplement: Supplementary file 1 [file Data_Sheet_1.PDF]

SUPPLEMENTAL MATERIALS. FOR ONLINE PUBLICATION WITH

The diel disconnect between cell growth and division in *Aureococcus* is interrupted by giant virus infection

Alexander R. Truchon[1†], Emily E. Chase[1†], Ashton R. Stark[1], Steven W. Wilhelm[1*]

[1]Department of Microbiology, University of Tennessee, Knoxville, TN 37996, United States

Key Words: diel periodicity, harmful algal bloom, *Nucleocytoviricota*, host-virus system, virocell

[†]These authors contributed equally.

[*]Corresponding author.

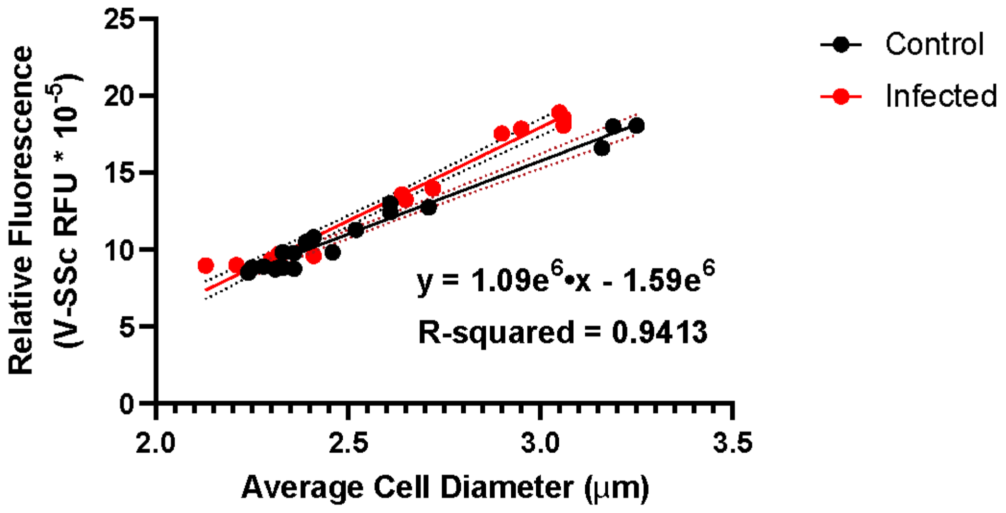

Figure S1. Correlation between violet side-scatter measurements taken on the Cytoflex flow cytometer and average cell diameter measured *via* FlowCam.

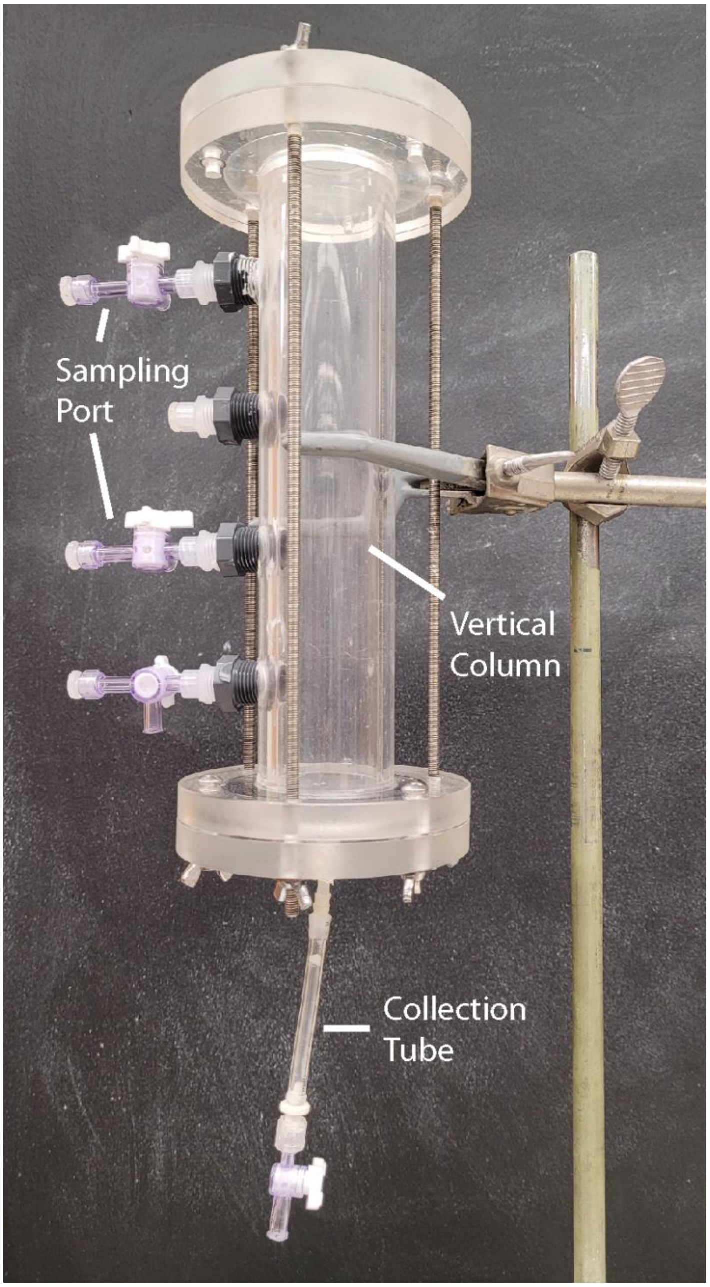

Figure S2. The setup of the vertical sinking column used in this study.

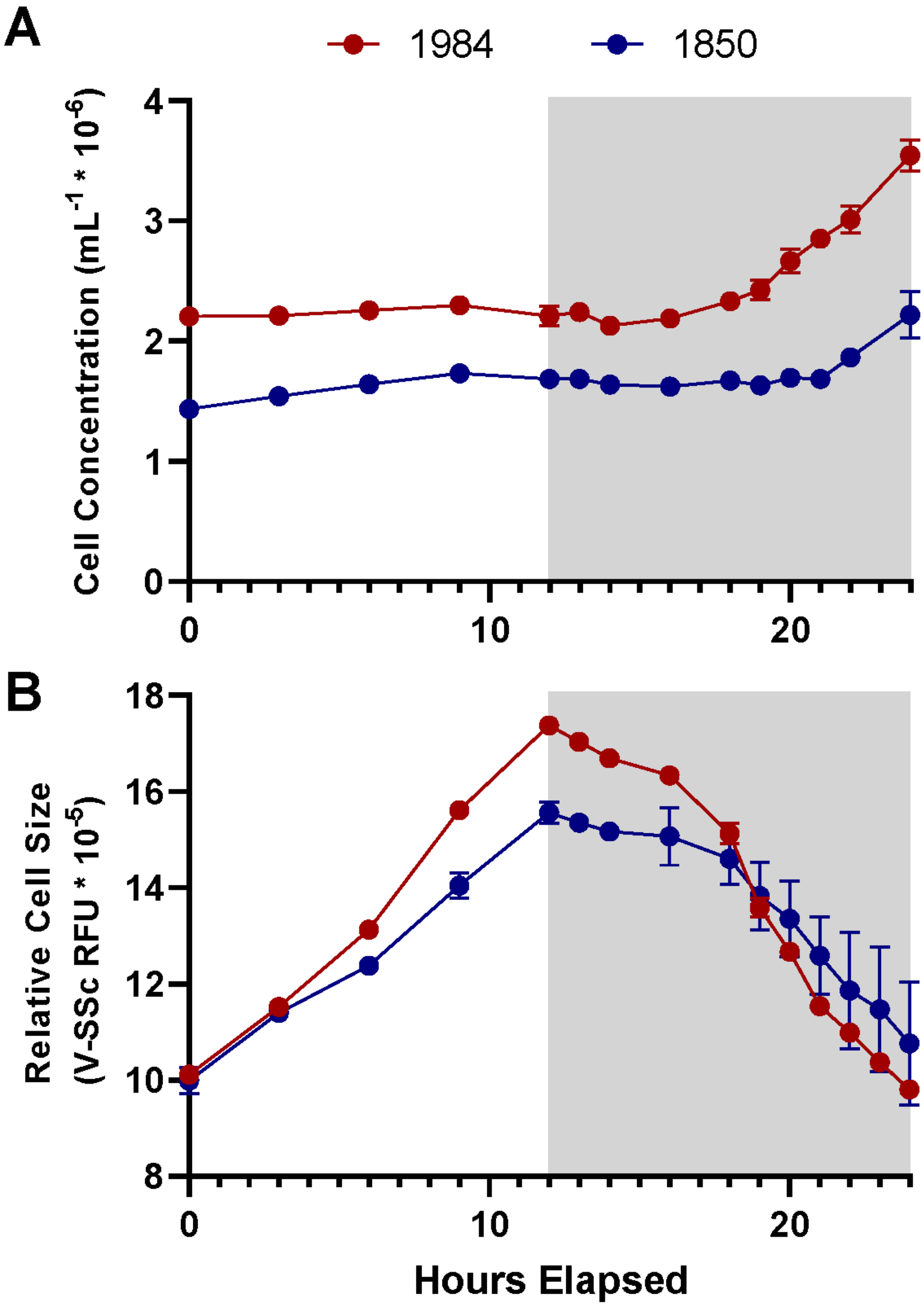

Figure S3. Cell density (A) and approximate cell diameter (B) of two *A. anophagefferens* strains, CCMP1984 (red) and CCMP1850 (blue) with particular attention paid to the night period.

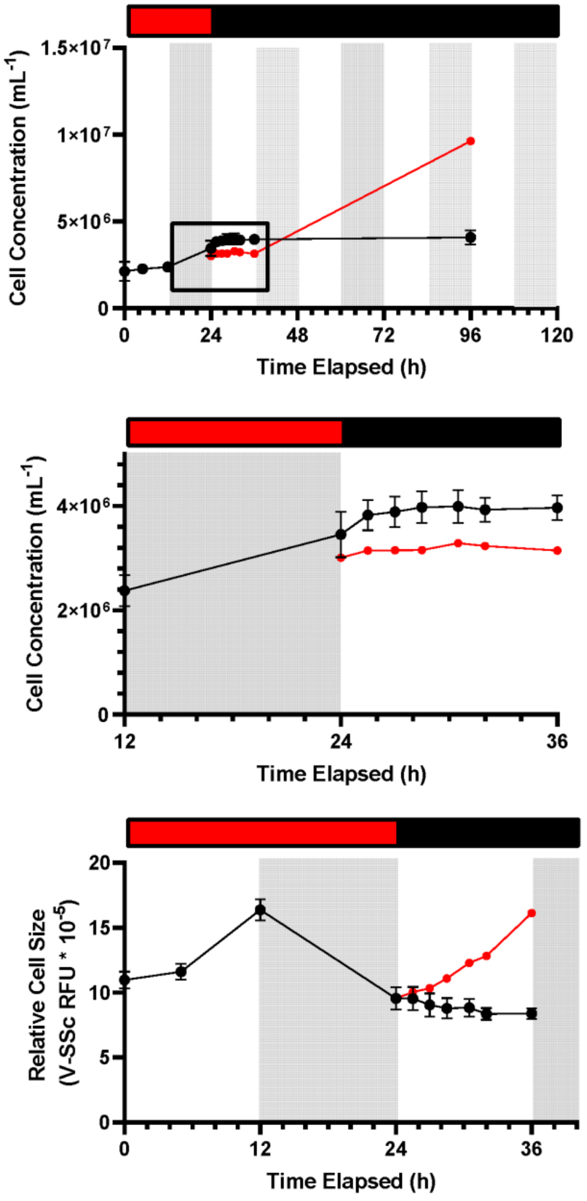

Figure S4. Free-running clock experiments on *A. anophagefferens*. Cultures were entrained to a consistent diel cycle before and either left in the same cycle at the start of the light period (red) or moved to a parallel fully dark incubator (black) at the 24-hour timepoint. Cell concentrations were measured frequently in the first 12 hours of the free-running clock (A) after 96 hours (B) either in complete darkness or in the regular diel cycle. Cell diameter was also measured within the first 12 hours of the free-running clock (C). Grey and white backgrounds only refer to the light cycle of the entrained samples while red and black periods above the graphs refer to the entrainment period and the free-running clock.

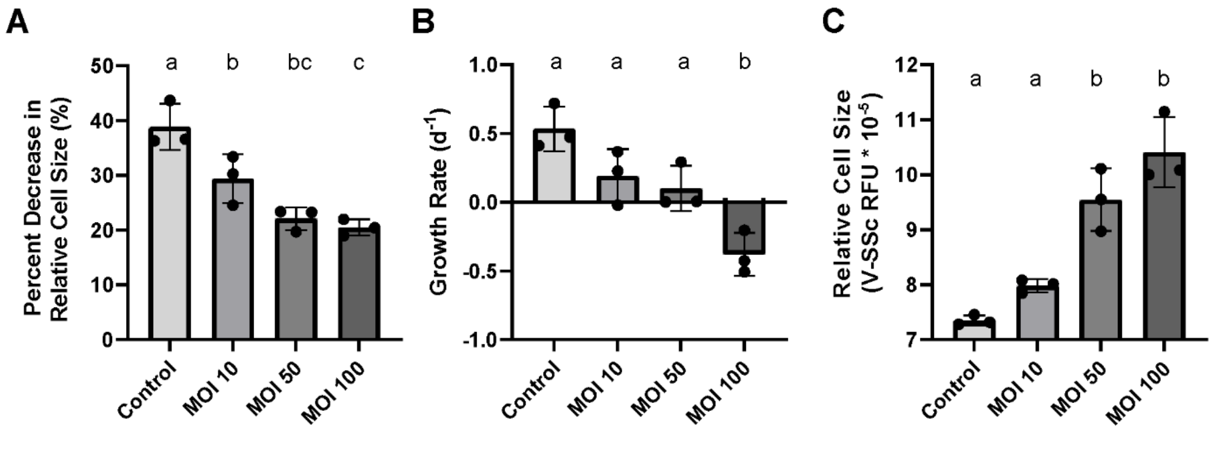

Figure S5. (A) Effect of decreasing M.O.I. on the average cell diameter in cultures 24 hours after infection. (B-E).

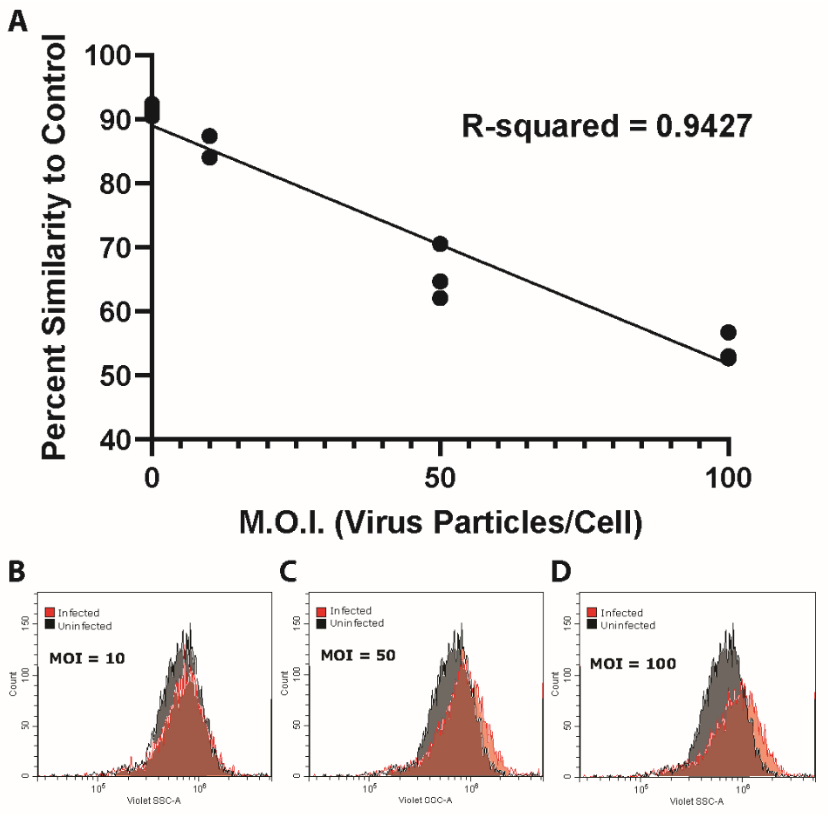

Figure S6. Percent similarity between control samples and infected treatment samples were calculated *via* the overlay function in the Cytoflex software CytExpert on the violet side-scatter channel using a violet laser (A). Correlation between percent similarity to the control after 23 hours of infection and MOI is denoted (n = 3). Cytograms detailing the overlap of individual control samples and samples of each MOI are shown (B-D).

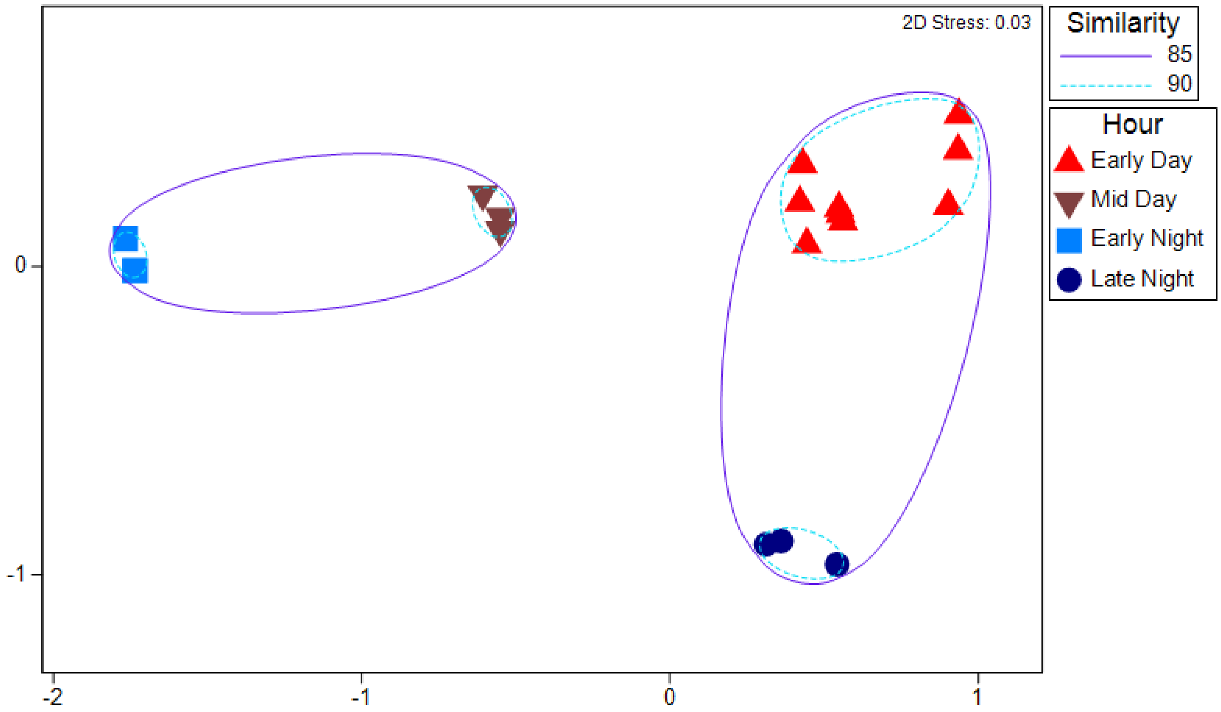

Figure S7. NMDS analysis of all uninfected samples colored based on the time of day they were taken.

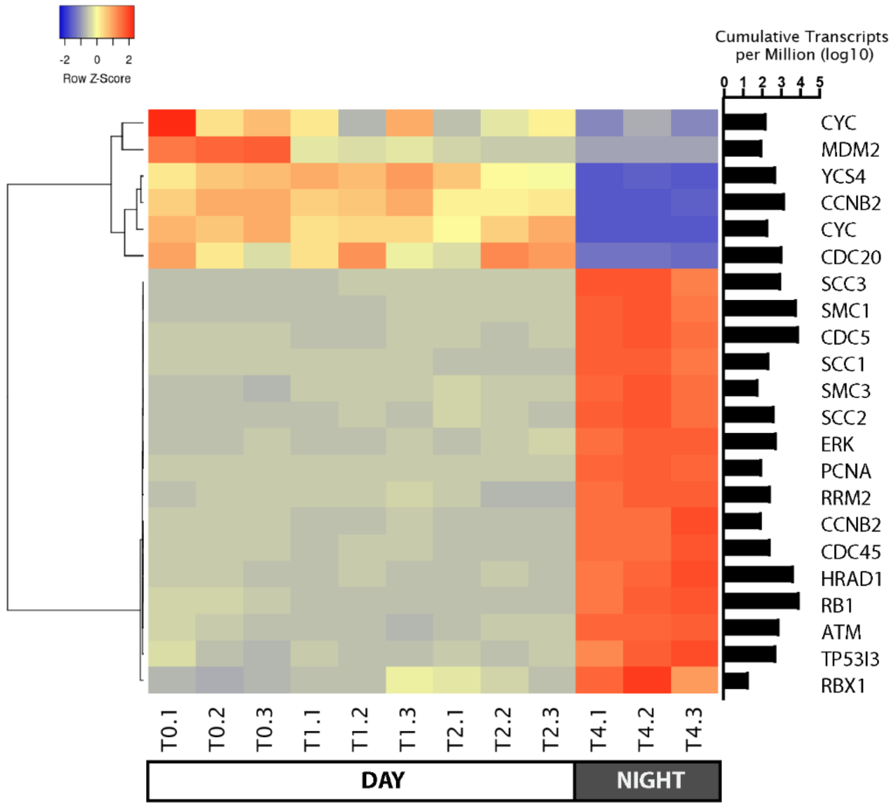

Figure S8. Heatmap of DESeq2 normalized cell cycle gene read counts that were found to be differentially expressed (p-value < 0.05, log2fold change > 2 or < -2) between the early day and the early night control transcriptome samples. The sum of transcripts across all treatments for each individual gene are indicated in log(TPM).

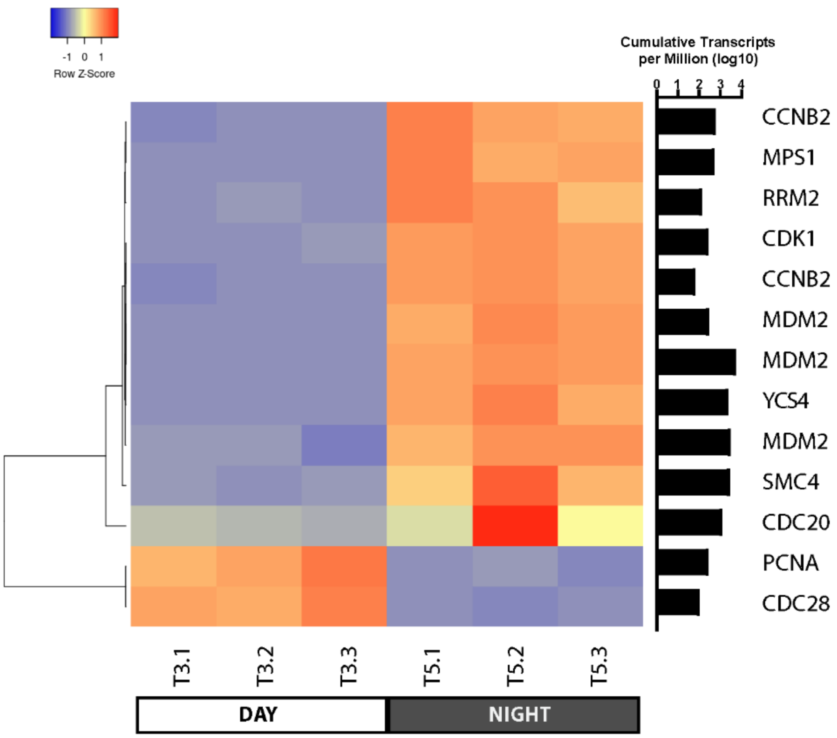

Figure S9. Heatmap of DESeq2 normalized cell cycle gene read counts that were found to be differentially expressed (p-value < 0.05, log2fold change > 2 or < -2) between the late day and late night control transcriptome samples. The sum of transcripts across all treatments for each individual gene are indicated in log(TPM).

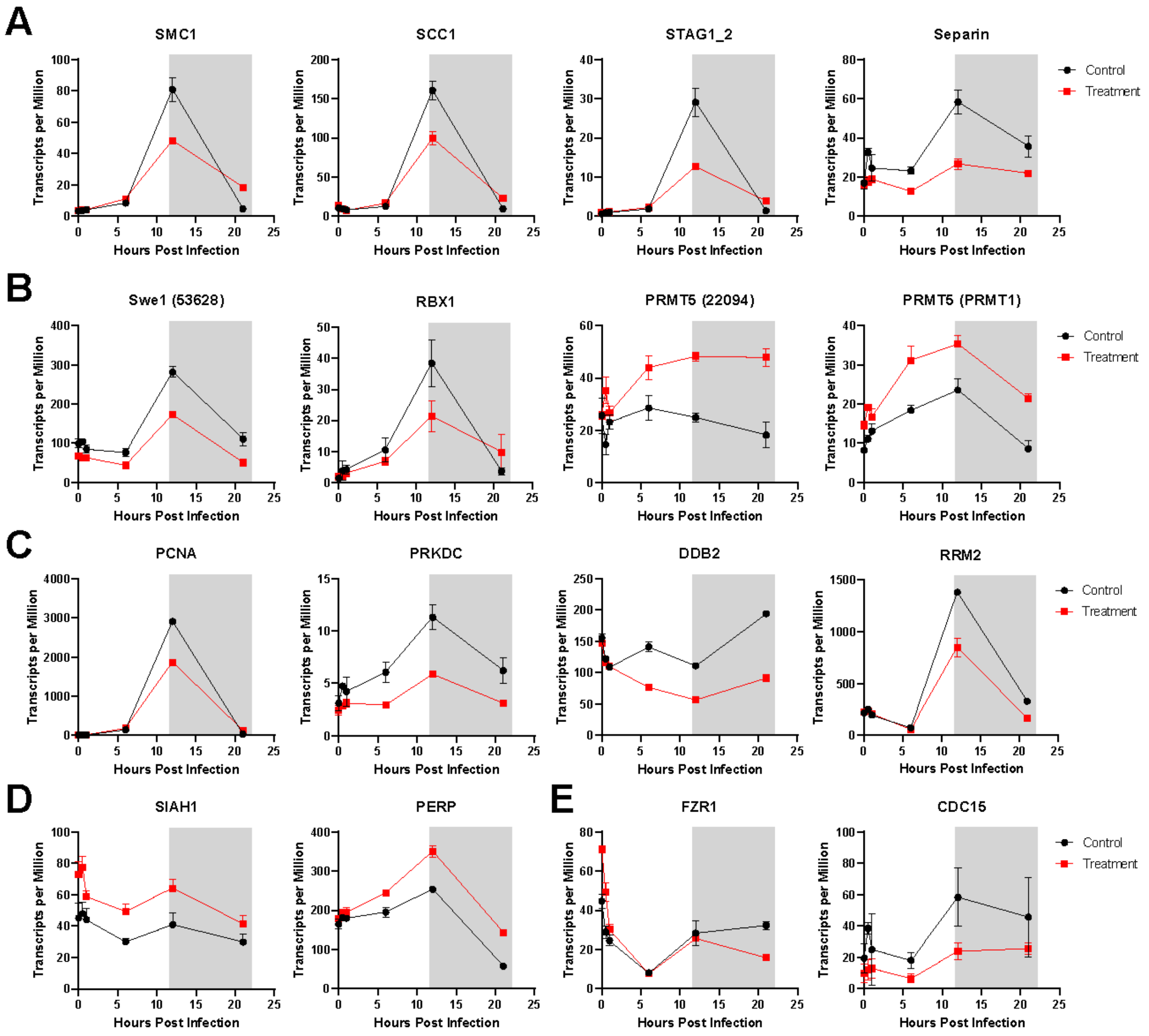

Figure S10. A selection of differentially expressed genes between and their relative expression patterns (TPM) throughout the infection cycle between control uninfected (black) and infected (red) treatments. (A) Expression of cohesin associated genes, (B) Swe1 and several of its regulatory genes, (C) genes involved in DNA replication and regulation of DNA damage, (D) apoptosis associated genes, and (E) drivers of mitotic division.

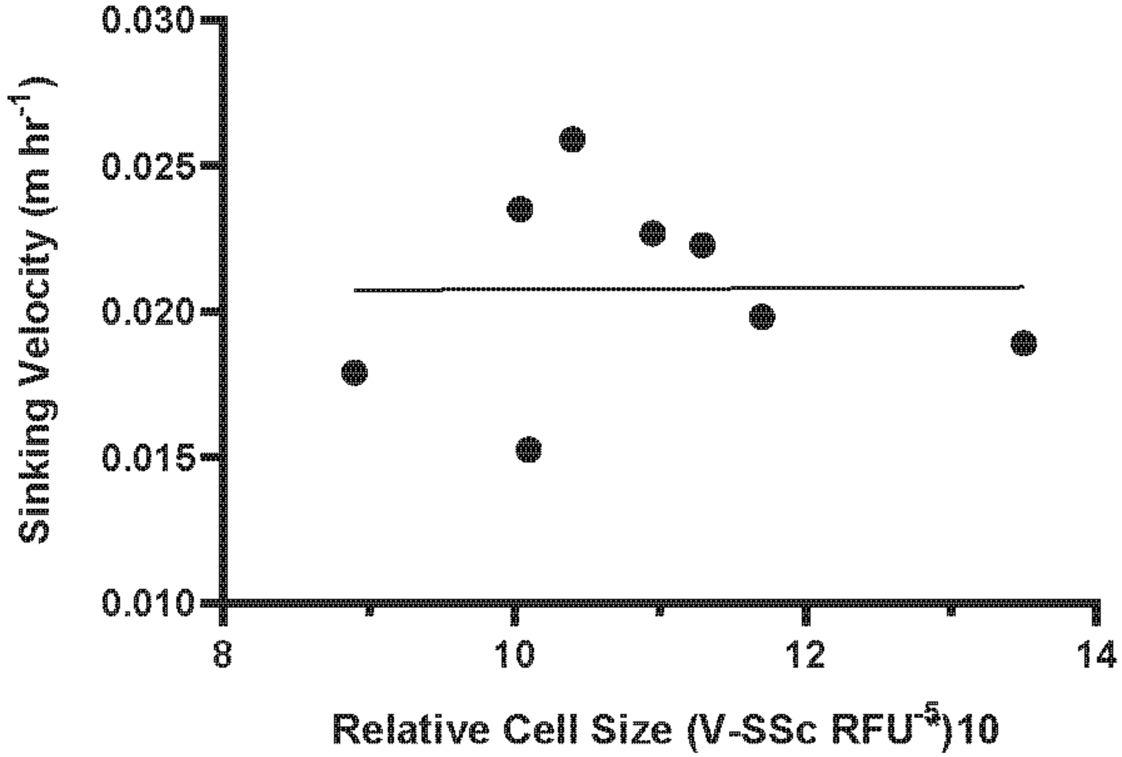

Figure S11. Correlation between relative fluorescence (SSc) and sinking velocity of *A. anophagefferens* CCMP1984 cells.

Table S1. Growth dynamics of *A. anophagefferens* in the presence of either total light or total dark. Rates are given ± S.D.

	CCMP1707		CCMP1850		CCMP1984	
	Dark	Light	Dark	Light	Dark	Light
**Growth Rate @24 hr (d^{-1})**	0.016 (0.001)	-0.095 (0.077)	0.029 (0.028)	-0.118 (0.017)	0.002 (0.020)	-0.021 (0.028)
**Growth Rate @48 hr (d^{-1})**	0.012 (0.020)	-0.147 (0.116)	0.022 (0.021)	-0.094 (0.016)	0.007 (0.013)	0.017 (0.055)
**% Change in Cell Size (@24 hr)**	-4.35 (1.81)	7.08 (2.98)	-8.45 (0.60)	5.45 (1.15)	-0.282 (4.21)	22.60 (2.62)
**% Change in Cell Size (@48 hr)**	-18.30 (2.92)	16.10 (5.18)	-17.23 (1.42)	4.61 (0.45)	-12.27 (1.63)	34.50 (5.56)

Table S2. Gene set enrichment analysis detailing enrichment of cell cycle genes between time points of uninfected transcriptomes. Columns represent time points as dependent variables and rows represent time points as independent variables, with positive values corresponding to increased representation (transcript abundance) of the dependent variable and negative values representing decreased representation. The "rest" variables represent the testing of one time point against all other time points. Nominal p-values testing whether the gene set is significantly enriched between time points are displayed in parentheses.

	Early Day	Late Day	Early Night	Late Night	Rest
**Early Day**		1.18 (0.17)	1.87 (0.0)	1.35 (0.0)	1.28 (0.19)
**Late Day**	-1.20 (0.15)		1.55 (0.0)	1.27 (0.12)	1.04 (0.45)
**Early Night**	-1.88 (0.0)	-1.54 (0.0)		-1.73 (0.0)	-1.81 (0.0)
**Late Night**	-1.36 (0.0)	-1.29 (0.09)	1.73 (0.0)		-0.79 (0.78)
**Rest**	-1.29 (0.18)	-1.05 (0.42)	1.80 (0.0)	0.80 (0.79)	

Table S3. Counts of differentially expressed genes between any two time points, between night and day, early night and early day, and late night and late day. Fractions of all genes (11,706 for all genes and 170 for cell cycle genes) and all differentially expressed genes are represented as percentages.

	**Any Time Points**		**Night V. Day**		**Early Night V. Early Day**		**Late Night V. Late Day**	
	**All Genes**	**Cell Cycle Genes**	**All Genes**	**Cell Cycle Genes**	**All Genes**	**Cell Cycle Genes**	**All Genes**	**Cell Cycle Genes**
**DE Genes**	1823	35	1683	30	1220	22	520	13
**% of Total Genes**	15.57	20.59	14.38	17.65	10.42	12.94	4.44	7.65
**% of DE Genes**	NA	1.92	NA	1.78	NA	1.80	NA	2.50

Table S4. All *A. anophagefferens* cell-cycle associated genes which are either upregulated or downregulated 1.5-fold (p-value < 0.05) at either the 6-hour, 12-hour, or 24-hour timepoints in the presence of *K. quantuckense.*

**Gene_ID**	**Gene**	**6-hour (log2-fold change)**	**12-hour (log2-fold change)**	**21-hour (log2-fold change)**	**Notes**
**Cohesin, Condensin, and Other Associated Genes**					
AURANDRAFT_64106	SCC1	NA	Down (-0.64)	Up (1.14)	*Cohesin complex subunit 1*
AURANDRAFT_70503	SMC1	NA	Down (-0.69)	Up (1.81)	*Structural maintenance of chromosome 1; Cohesin subunit*
AURANDRAFT_72635	SMC3	NA	NA	Up (1.55)	*Structural maintenance of chromosome 3; Cohesin subunit*
AURANDRAFT_60440	STAG1_2	NA	Down (-1.15)	Up (1.34)	*Cohesin complex subunit SA-1*
AURANDRAFT_26480	SCC2	Down (-0.94)	Down (-1.49)	Down (-1.39)	*Cohesin loading factor subunit 2*
AURANDRAFT_63990	SCC2	NA	NA	Up (1.43)	*Cohesin loading factor subunit 2*
AURANDRAFT_36910	SMC2	NA	Up (0.75)	Up (1.84)	*Structural maintenance of chromosome 2; Condensin subunit*
AURANDRAFT_72033	SMC4	NA	Down (-1.02)	Down (-1.50)	*Structural maintenance of chromosome 4; Condensin subunit*
AURANDRAFT_65310	YCS4	NA	Down (-0.80)	Down (-1.44)	*Condensin complex subunit 1*
AURANDRAFT_72664	ESP1;NDC80	Down (-0.89)	Down (1.07)	Down (-0.87)	*Separin*
**Swe1 Regulation**					
AURANDRAFT_53628	Myt1;Wee1	Down (-0.84)	Down (-0.65)	Down (-1.30)	*Mitosis inhibitor protein kinase*
AURANDRAFT_71641	Myt1;Wee1;Swe1	NA	Down (-0.92)	Down (-1.31)	*Mitosis inhibitor protein kinase*
AURANDRAFT_72516	Myt1;Wee1;Swe1	NA	Down (-0.94)	Down (-1.75)	*Mitosis inhibitor protein kinase*
AURANDRAFT_22094	PRMT5	Up (0.60)	Up (1.01)	Up (1.24)	*Type II protein arginine methyltransferase; Swe1 regulation*
PRMT1	PRMT5	Up (0.74)	Up (0.64)	Up (1.16)	*Type II protein arginine methyltransferase; Swe1 regulation*
AURANDRAFT_22764	RBX1	Up (0.79)	NA	Up (1.19)	*E3 ubiquitin-protein ligase; Swe1 regulation*
AURANDRAFT_8889	RBX1	NA	Down (-0.80)	Up (1.31)	*E3 ubiquitin-protein ligase; Swe1 regulation*
AURANDRAFT_58667	SKP1	NA	NA	Up (1.82)	*S-phase kinase-associated protein 1; Swe1 regulation*
AURANDRAFT_52007	CUL1	NA	NA	Up (0.80)	*Cullin 1; Swe1 regulation*
AURANDRAFT_55274	CUL1	NA	Down (-0.58)	NA	*Cullin 1; Swe1 regulation*
AURANDRAFT_4905	CDC5	NA	Down (-1.17)	NA	*Cell division control protein 5; Swe1 regulation*

Table S2 Continued
Apoptotis Associated Genes

AURANDRAFT_28360	CYC	NA	Down (-0.67)	NA	*Cytochrome C*
AURANDRAFT_59926	CYC	Up (0.72)	NA	Up (1.41)	*Cytochrome C*
AURANDRAFT_31918	PERP	NA	NA	Up (1.16)	*TP53 apoptosis effector proteins*
AURANDRAFT_28417	TP53I3	NA	NA	Up (0.65)	*P53-inducible protein 3*
AURANDRAFT_20166	SIAH1	Up (0.69)	Up (0.71)	NA	*E3 ubiquitin-protein ligase; methyltransferase domain*
AURANDRAFT_72557	LRDD	NA	NA	Down (-0.97)	*Death domain-containing protein*
**p53 Negative Feedback**					
AURANDRAFT_12504	MDM2	Down (-1.23)	Up (2.79)	NA	*E3 ubiquitin-protein ligase; p53 regulation*
AURANDRAFT_24297	MDM2	Down (-0.97)	Down (-1.72)	Down (-1.03)	*E3 ubiquitin-protein ligase; p53 regulation*
AURANDRAFT_27943	MDM2	NA	Down (-1.18)	Down (-0.74)	*E3 ubiquitin-protein ligase; p53 regulation*
AURANDRAFT_3154	MDM2	Down (-0.85)	Down (-1.51)	Down (-1.03)	*E3 ubiquitin-protein ligase; p53 regulation*
AURANDRAFT_33333	MDM2	NA	Down (-0.89)	NA	*E3 ubiquitin-protein ligase; p53 regulation*
AURANDRAFT_72197	MDM2	NA	Down (-0.63)	NA	*E3 ubiquitin-protein ligase; p53 regulation*
AURANDRAFT_63280	PPM1D	Down (-0.77)	Down (-1.00)	Down (-1.08)	*Protein phosphatase 1D; p53 regulation*
**Regulation of DNA Replication**					
AURANDRAFT_70336	MCM3	NA	Down (-0.79)	NA	*DNA replication licensing factor 3*
AURANDRAFT_63773	MCM4	Up (0.71)	NA	NA	*DNA replication licensing factor 4*
AURANDRAFT_33815	MCM4	NA	Down (-0.60)	NA	*DNA replication licensing factor 4*
AURANDRAFT_38450	MCM5	NA	Down (-0.75)	Down (-0.68)	*DNA replication licensing factor 5*
AURANDRAFT_24050	MCM6	NA	Down (-0.59)	NA	*DNA replication licensing factor 6*
AURANDRAFT_53352	MCM7	NA	Down (-0.84)	Down (-0.81)	*DNA replication licensing factor 7*
AURANDRAFT_72178	MCM7	NA	Down (-0.85)	NA	*DNA replication licensing factor 7*
AURANDRAFT_63471	ORC2	NA	Down (-0.67)	Down (-0.62)	*Origin recognition complex subunit 1*
AURANDRAFT_70163	PCNA	NA	NA	Up (1.77)	*Proliferating cell nuclear antigen*
AURANDRAFT_62055	CDC6	NA	NA	Down (-1.17)	*Cell division control protein; DNA replication-associated*
AURANDRAFT_20277	CDC7	NA	NA	Up (0.62)	*Cell division control protein; DNA replication-associated*
AURANDRAFT_71439	DDB2	Down (-0.91)	Down (-0.91)	Down (-1.25)	*DNA damage-binding protein 2*
AURANDRAFT_68528	PRKDC	Down (-1.09)	Down (-0.90)	Down (-1.18)	*DNA-dependent protein kinase*

RRS3	RRM2	NA	Down (-0.64)	Down (-1.18)	*Ribonucleoside-diphosphate reductase M2*
AURANDRAFT_64915	CCDN2	NA	Up (0.58)	NA	*G1/S-specific cyclin D2*
**Regulation/Termination of Mitosis**					
AURANDRAFT_22946	APC7	NA	NA	Up (0.61)	*Anaphase-promoting complex subunit 7*
AURANDRAFT_28025	APC7	NA	NA	Up (0.62)	*Anaphase-promoting complex subunit 7*
AURANDRAFT_28134	APC7	NA	Down (-0.63)	NA	*Anaphase-promoting complex subunit 7*
AURANDRAFT_9374	APC11	Up (0.77)	NA	NA	*Anaphase-promoting complex subunit 11*
AURANDRAFT_28425	FZR1	NA	NA	Down (-1.18)	*Cell division cycle 20 protein; cofactor of APC complex*
AURANDRAFT_12473	CCNB2	NA	NA	Down (-0.94)	*G2/mitotic-specific cyclin B2*
AURANDRAFT_38949	CCNB2	NA	NA	Down (-0.75)	*G2/mitotic-specific cyclin B2*
AURANDRAFT_64231	CCNB2	NA	NA	Down (-1.37)	*G2/mitotic-specific cyclin B2*
AURANDRAFT_71534	CCNB2	NA	NA	Down (-0.59)	*G2/mitotic-specific cyclin B2*
AURANDRAFT_31094	CDK1	NA	NA	Down (-0.97)	*Cyclin-dependent kinase 1; G2 to M-phase*
AURANDRAFT_13918	TTK/MPS1	NA	NA	Down (-0.88)	*Monopolar spindle kinase*
AURANDRAFT_19390	MAD2	NA	NA	Down (-0.75)	*Mitotic spindle assembly checkpoint protein*
AURANDRAFT_70730	HRAD1	NA	NA	Up (0.88)	*Cell cycle checkpoint protein*
AURANDRAFT_71576	PTEN	NA	Down (-0.95)	Down (-1.23)	*Phosphatase and tensin homolog; tumor supression*
AURANDRAFT_32347	TEM1	NA	NA	Up (1.07)	*GTP-binding protein; termination of M-phase*
AURANDRAFT_32157	CDC15	Down (-1.51)	Down (-1.23)	NA	*Protein kinase; cell division control protein*
**Other Cell Cycle-Associated Genes**					
AURANDRAFT_30303	ERK;MAPK1_3	NA	NA	Down (-1.09)	*Mitogen-activated protein kinase*
AURANDRAFT_31374	ERK/MAPK1_3	NA	NA	Up (0.79)	*Mitogen-activated protein kinase*
AURANDRAFT_39527	ERK/MAPK1_3	NA	NA	Down (-0.63)	*Mitogen-activated protein kinase*
AURANDRAFT_55239	ERK/MAPK1_3	Up (0.59)	NA	Up (1.21)	*Mitogen-activated protein kinase*
CDK1	CDC28/CDC2	NA	Down (-0.73)	NA	*Cyclin-dependent kinase*
AURANDRAFT_58854	CDK7	NA	NA	Up (0.87)	*Cyclin-dependent kinase 7*
AURANDRAFT_69594	CCNH	NA	NA	Up (0.67)	*Cyclin H*
AURANDRAFT_12637	RB1	NA	NA	Up (1.24)	*Retinoblastoma-associated protein-like; ribonuclease H2*
AURANDRAFT_68494	GSK3B	Down (-0.97)	NA	NA	*Glycogen synthase kinase 3*
